# Supplementary material for: The impact of school absence on mental health in children and young people: Analysis of an English national birth cohort
Source: PLoS One. 2026 Jan 14;21(1):e0336137. doi: 10.1371/journal.pone.0336137 (PMC12803469; doi:10.1371/journal.pone.0336137)
Supplement: Attachment — Submitted filename: Response reviewers PlosOne absence MH 040225.docx [file pone.0336137.s002.docx]

Response to reviewers

Dear Editor

Thank you for asking us to revise this paper. We respond point by point to the Editorial and reviewer comments below

Russell Viner on behalf of all authors

Editorial comments:

1. ***Disentangle school closures and absenteeism:*** *The manuscript conflates these concepts, which have distinct implications, and we recommend clarifying their differences.*

Response: We agree these are distinct and have distinct implications, although of course in both children are not in school. We have rewritten and restructured the introduction and discussion to emphasize that we are interested in school absence (i.e. absenteeism), but also to recognise that in the current post-pandemic generation these impacts can be difficult to separate from the forced absence due to school closures during the pandemic. Therefore we argue that examining pre-pandemic data can be helpful to ensure these impacts are distinct. We have removed any suggestion that these data might be helpful in understanding impacts of school closures, although we note that in future pandemics there may be very high rates of absenteeism while schools are open, due to parental anxiety, and that understanding such impacts upon mental health will be important for policy.

2. ***Endogeneity concerns in the causal relationship:*** *Despite controlling for maternal mental health, the bidirectional relationship between absenteeism and mental health raises concerns that should be acknowledged.*

Response: We undertook non-lagged and ‘lagged’ analyses specifically to account for prior associations between mental health and absence. Because of the temporal aspects of these variables, this did not require a directionality to the relationship i.e. it adjusted for the relationship at earlier periods regardless of directionality. We recognise that our DAG implied a directionality to this – although this is required of directed acyclic graphs. However our models through our DAG did allow us to account for previous bi-directional relationships between mental health and absence at earlier times. However we recognise in our revised limitations sections that residual confounding may remain.

3. ***Mechanisms linking absenteeism to mental health:*** *The manuscript could benefit from a brief discussion on how absenteeism disrupts key benefits provided by schools, such as structure, social interaction, and essential services.*

Response: We have included a brief discussion of this as suggested in the first paragraph of the revised introduction.

Review comments.

Reviewer #1

*1. Re equating school absence with school closure*

Response: see response to Editorial comment 1 above

*2. Handling of missing data; use of attrition weights.*

Response: Our analyses relate to a subset of the MCS for which linked educational data were available. This is a subset, albeit a large subset, of the overall dataset. It is therefore not a more standard MCS analysis. It is difficult to imagine on what basis one might multiply impute this linked dataset, as we note in our current limitations section that “Not all MCS participants in England had linked educational data, likely reflecting loss during the linkage process and lack of consent for linkage. It is likely these data are missing at random as non-linkage is unlikely to be related to any of the variables under study here.”

Similarly, attrition weights are not available for this linked dataset and we believe it would be incorrect to use weights for non-linked datasets. We note in our current limitations section that “There was minor excess attrition in the sample for cohort members from the most deprived deciles and from some ethnic groups; whilst this is unlikely to impact the substantive relationship between school absence and mental health problems, the association of deprivation with both absence and mental health problems suggest our findings may under-estimate its size.”

*3. elaborate on their use of the marginal structural model (MSM) and its advantages compared to standard regression techniques*

Response: We cite a number of papers in our methods section that outline the advances of MSM methods compared to standard regression techniques, and our Methods and What this study adds sections provide considerable detail on the advantages of these methods. We believe that additional detail is beyond the scope of this paper.

*4. unobserved confounding*

Response: we have strengthened our limitations section (as above) to better recognise the potential for unobserved confounding.

*5. frame the results as robust associations rather than definitive causal effects*

Response: We agree and have modified our language to conclude we have shown ‘strong and potentially causal’ associations using causal inference methods.

Reviewer 2.

*1. issues about school absence and lockdown.*

Response: see response to Editorial comment above

*2. various typos and missing words*

Response: Thank you, these have been corrected.

*3. access to NPD*

Response: We have added a note that “These data are available on special application through the UK Data Service Secure Lab (<https://ukdataservice.ac.uk/help/secure-lab/)>.”

*4. quartiles?*

Response: We explain in our current methods section that we chose a categorical analysis as the association was very unlikely to be linear. This proved to be the case e.g. the medians were 0 as in Table 1. We chose quartiles as we wished to identify which sections of the child population were at risk, whether this was only those more extreme (i.e. highest quartile).

*5. selection of confounding variables?*

Response: We have revised our methods section to note “Our choice of hypothesized confounding variables was guided by the literature noted above.”

*6. total absence sessions for 11 year olds?*

Response: we had inverted the mean and the low confidence interval – thank you, this has been corrected.

*7. representativeness in terms of absence?*

Response: We do not have this information, yet this is a very large sample with linkage to routine administrative data, allowing the presumption of representativeness.

*8. bidirectional causal pathways?*

Response: see response to Editorial comments.

*9. lockdown versus school absence?*

Response: see response to Editorial comments.

*10. Thresholds of absence – 4 and 6%*

Response: we agree that the level of absence which appears to lead to mental health harms is lower than the UK definition of persistent absence at >10%. We have added the following to our conclusions: “This threshold is notably lower than the 10% threshold used to define persistent absence in England. Whilst higher levels of post-pandemic absence across the school population mean that quartiles of school absence will be different to those identified here, our findings suggests that mental health harms related to absence are likely at lower levels of school absence.”
