# Supplementary material for: The impact of school absence on mental health in children and young people: Analysis of an English national birth cohort
Source: PLoS One. 2026 Jan 14;21(1):e0336137. doi: 10.1371/journal.pone.0336137 (PMC12803469; doi:10.1371/journal.pone.0336137)
Supplement: Attachment — Submitted filename: Viner PlosOne response to reviewers 300925.docx [file pone.0336137.s003.docx]

Dear Editor

Thank you for asking us to revise and resubmit this article.

We respond to reviewers comments point by point below.

Reviewer #2: Thanks for addressing the review points and the tracked changes on the manuscript.
I think there could have been a little more emphasis on school attendance as an increased concern now compared to during and prior to the pandemic, with some indication of how figures have worsened, particularly the near doubling of persistent absence. But the point around attendance as policy concern and caution around causation is now clear and this is important given the propensity for assuming poor mental health causes absence on less robust data and analysis than this.

Minor point for proofs, “Persistently absence” in the abstract should be “persistent absence”

**Response**: Corrected

Reviewer #3: The study „The impact of school absence on mental health in children and young people: analysis of an English national birth cohort“ uses inverse probability weighting and linked administrative and longitudinal survey data to estimate the effect of school absences on mental health. The study addresses an important topic, makes an important contribution to the literature, and uses high-quality data and a rigorous method. Nonetheless, there are a few points that need to be addressed before publication.

1. I suggest that the authors describe the method they used in the final sentence of the introduction, rather than stating that they “estimated the robust effect of school absence on mental health.” I recommend wording such as: “We use marginal structural models and control for a large set of plausible confounders to estimate the causal effect […]”.

**Response**: Thank you. We have edited the final sentence of the introduction as follows:
“We therefore used a nationally representative UK birth cohort with linked routine education administrative data to estimate the effects of school absence on mental health using marginal structural models and controlling for a wide set of plausible confounders. We hypothesized that higher absence increased the risk of later mental health problems.”

2. The authors should describe the dataset in more detail. For example, it should be noted that the MCS oversampled children from areas with a high proportion of ethnic minorities and from economically disadvantaged areas. Additionally, it should be mentioned that the NPD only contains data on students attending public schools.

**Response**: Thank you. We have provided additional data as suggested in the revised Methods section:

“The MCS oversampled children from economically disadvantaged areas and areas with a high proportion of ethnic minorities.”

And

“Routine educational data on school absence and educational attainments from the National Pupil Database (NPD)[16] were linked to MCS participants in England only, with data only available for those attending public schools.”

3. The authors should explicitly state that they did not use any survey weights, which is usually suggested for MCS data.

**Response**: Thank you. We have added the following to our revised Analysis section:

“We did not use population weighting methods provided for MCS analyses as these are not applicable for analyses across multiple waves as we have undertaken.”

4. Please clarify exactly how many cases were excluded due to complete case analysis. Also, indicate how frequently missing values were replaced using data from the previous sweep.

**Response**: We note in the current paper that “The sample for these analyses consists of those children with data on both school absence and mental health disorder at each age: n=7659 at 7 years, n=6689 at 11 years, n=5838 at 14 years.” The sample sizes for each confounding variable are shown in Table 2 and the final samples for the models are shown in Tables 3-4. We have included a line on sample sizes for the marginal structural models compared with the analytic sample as follows:

“Final model samples were 6383 at 7 years (83.3% of the analytic sample at 7 years), 6102 at 11 years (91.2% of the analytic sample) and 5616 at 14 years (96.2% of analytic sample).”

Re replacement using data from the previous sweep, this referred to whole variables rather than individual-level missing data, i.e. where data on a confounding variable was present at 7 years but not at 11 years, the 7 year data was used as a confounder for the 14 year models. We have clarified this in the revised Methods section as follows:

“Where a required confounding variable was not available at a wave, the relevant variable from the preceding wave was substituted to ensure temporal precedence was maintained.”

5. Figure 1 does not include an arrow from C to Ab9&10 and Ab12&13. Was this omission intentional? Is C included in all models?

**Response**: Thank you. C was included in all models. As C was only included once in the lagged models we omitted the arrows from C to Ab9&10 and Ab12&13. We feel it is better to note this in the legend for Figure 1 rather than provide additional arrows.

We have edited the Figure 1 legend as follows:

“Note that C baseline confounders were included in each of the models, including for Ab9&10 and Ab12&13 however only a single arrow is shown in Figure 1 for simplicity and as C confounders were only included once in lagged models.”

6. Why do the authors rely on parent-reported SDQ scores rather than self-reported SDQ scores? Are the results robust when using self-reported SDQ scores?

**Response**: Only parent-reported SDQ scores are available at 7, 11 and 14 years so we used these for consistency.

7. I do not understand why the authors present the results of the “unlagged model” in the abstract. Their DAG clearly shows that lagged absences and lagged mental health are confounders of the association between current absences and current mental health. The authors also acknowledge this in the discussion: “Estimates for lagged models for 11 and 14 years were smaller than in unlagged models, suggesting that failing to account for confounding histories likely results in upwards-biased estimates.” Therefore, if the authors aim to estimate the causal effect, they should focus exclusively on the results of the lagged model.

**Response**: Thank you for this. We recognise that the Abstract currently mixes unlagged data (for highest quartile) and lagged data (for persistent absence). We agree and have modified the Abstract to report only the lagged models for 11 and 14 years (there is no lagged model for 7 years).

8. The authors need to provide a more detailed explanation of how they selected potential confounders. Their aim is to estimate the causal effect of absences on mental health, which can be achieved with marginal structural models only if all relevant confounders are included. Therefore, simply stating, “Our choice of hypothesized confounding variables was guided by the literature noted above,” is not sufficient. I recommend that the authors consider the risk factors for absences summarized in the meta-analysis by Gubbels et al. (2019), as many of these factors may also influence mental health.

**Response**: We carefully considered a range of potentially confounding factors for the mental health and absence associations as discussed in the literature reviewed in the Background for the paper. We followed good practice in identifying a minimally sufficient confounder set, recognising the importance of parsimony and avoiding collider bias. We thank the reviewer for their suggestion and confirm that potential confounders or other similar proxies were considered.

9. In addition, I believe important confounders are missing, such as the socio-economic status of the family (which affects absences independently of area-level deprivation; Klein et al., 2020).

**Response**: We recognise the multidimensionality of deprivation, as outlined in the suggested reference (Klein). Very small area measures, such as the English indices of multiple deprivation (IMD), provide proxies for multiple dimensions of deprivation. Again the importance of parsimony in deciding a minimally sufficient confounder set was important to us here. We elected to only use one multi-dimensional measure of deprivation, although we recognise that there will necessarily be some small degree of variation in individual measures within a very small area measure. We believe it very unlikely that not additionally controlling for another measure of SES, such as income, as well as IMD, would leave open backdoor paths through which confounding may occur.

10. I do not fully understand the following sentences in the “limitations” section: “Not all MCS participants in England had linked educational data, likely reflecting loss during the linkage process and lack of consent for linkage. It is likely these data are missing at random as non-linkage is unlikely to be related to any of the variables under study here.”

First, it should be noted that the NPD does not contain information on students attending private schools. Second, do you mean “missing completely at random” (MCAR) or “missing at random” (MAR)? MCS-NPD linkage is related to demographics and socio-economic status, but conditional on these factors, it is not associated with internalizing and externalizing problems (Dräger et al. 2024, Appendix S1).

**Response**: As above, we have included a note that the NPD only contains information on students in public schools. In terms of the missingness, we used the term missing at random as we acknowledge the point made by the reviewer, although we don’t believe it requires further revision of the document.
